# New transcription signals in SARS-CoV-2 reshape virus–host interactions

**DOI:** 10.1371/journal.pbio.3003744

**Published:** 2026-04-17

**Authors:** Isabel Sola, Sonia Zuñiga

**Affiliations:** Department of Molecular and Cell Biology, National Center of Biotechnology (CNB-CSIC), Darwin 3, Campus Universidad Autónoma de Madrid, Madrid, Spain

## Abstract

Non-spike changes driving SARS-CoV-2 fitness remain undiscovered. This Primer comments on two PLOS Biology papers that show that evolutionary N gene mutations create a transcription-regulating sequence producing a truncated N protein that enhances fitness by blocking antiviral responses.

## Teaser

Non-spike changes driving SARS-CoV-2 fitness remain undercharacterized. Two *PLOS Biology* papers show that evolutionary N gene changes create a transcription-regulating sequence producing a truncated N protein that enhances fitness by blocking antiviral responses.

Despite being among the largest known RNA viruses, coronaviruses have relatively small genomes—around 30,000 nucleotides—that must encode multiple essential functions for replication and interaction with the host. This requires highly compact genomes in which nucleotides not only encode proteins but also participate in RNA–RNA and RNA–protein interactions that regulate fundamental viral processes. Expression of structural and non-structural genes located at the 3′-end of the genome depends on the transcription of subgenomic mRNAs (sgmRNAs), which is driven by transcription-regulating sequences (TRSs). TRSs include a conserved core sequence (CS), typically 6–7 nucleotides long, and variable flanking sequences [1]. Sequence identity between the leader TRS at the 5′-end of the genome and TRSs preceding each gene is the main regulator of sgmRNA synthesis [2] and, consequently, of the expression of the encoded protein.

Since rapid sequence evolution is a hallmark of RNA viruses, the emergence of novel TRSs may lead to sgmRNAs encoding new proteins, introducing novel actors in virus–host interactions and reshaping viral replication and immune evasion. Deep sequencing has enabled the discovery of new sgmRNAs produced during infection [3]. However, functional studies are essential to determine their relevance in viral biology. Two recent *PLOS Biology* papers by Mears and colleagues and Mulloy and colleagues [3,4] characterized a novel sgmRNA transcribed from a TRS that emerged within the nucleocapsid (N) gene (TRS-N*) of SARS-CoV-2. A triple mutation (GGG→AAC) simultaneously generated a consensus CS motif (AAACGAAC) and two amino-acid substitutions (RG→KR) that modify N protein phosphorylation [5,6]. The novel sgmRNA encodes the C-terminal domain of the N protein, which includes a double-stranded RNA binding motif (dsRBM). Expression of a truncated form of the N protein (referred to here as N*; Mears and colleagues name it NiORF3 and Mulloy and colleagues, N*M210) via an internal sgmRNA represents a diversification mechanism that potentiates certain protein functions and increases complexity in virus–host interactions.

To dissect the biological relevance of nucleotide changes at both the RNA and protein levels, in both studies [3,4] recombinant viruses were engineered in which the presence of the internal TRS was uncoupled from amino-acid substitutions in the full-length N protein. Another critical aspect was evaluating the impact of these changes on viral fitness—a measure of survival efficiency across the virus life cycle. Growth–competition experiments were designed by co-infecting cells with equal amounts of two viruses until one displaced the other and became dominant in the population. Both Mulloy and colleagues [4] and Mears and colleagues [3] demonstrated that the TRS acquisition and the resulting higher levels of N* protein, rather than the amino acid substitutions, were the primary drivers of enhanced fitness in recombinant viruses engineered on the ancestral Wuhan backbone. These results are in line with a previous report [6] that also employed recombinant viruses on the ancestral backbone. By contrast, in the Alpha variant, Mears and colleagues [3] found that both the TRS and the amino-acid changes contribute to increase fitness, underscoring the importance of sequence context, as fitness reflects multiple interconnected viral factors. This raises questions about the physiological relevance of this particular genotype in new circulating variants that have accumulated additional mutations.

The new TRS-N* first appeared in early 2020 with lineage B.1.1 and persisted in its derived variants (Alpha, Gamma, and Omicron). The TRS-N* transiently disappeared in late 2024 because of an additional mutation at G28884C. Its reversion to the previous TRS-N* allowed it to dominate again in 2025 [4]. The repeated emergence of the TRS-N* and the evidence of convergent evolution [3] suggest that this change might confer an evolutionary advantage in humans. Nonetheless, its biological impact should be considered in the context of co-occurring mutations elsewhere in the genome, particularly those in the S gene, which are major drivers of evolution by increasing virus transmission and enabling escape from neutralizing antibodies.

The key question here was to identify the mechanisms underlying these phenotypic effects. Mears and colleagues hypothesized that the new N* protein, including the dsRBM, might antagonize the antiviral interferon (IFN) responses by sequestering cytoplasmic dsRNA and inhibiting activation of dsRNA cell sensors. Their functional studies confirmed a contribution of RIG-I sensing, since viruses lacking the TRS-N* were partially rescued in RIG-I knockout cells, but not in MDA5-deficient cells [3]. Interestingly, results from both Mears and colleagues and Mulloy and colleagues indicated that the enhanced fitness, associated with the presence of the TRS-N* and the resultant expression of the N* protein, was not only mediated by IFN antagonism, suggesting additional mechanisms for N* during infection.

**Fig 1:**
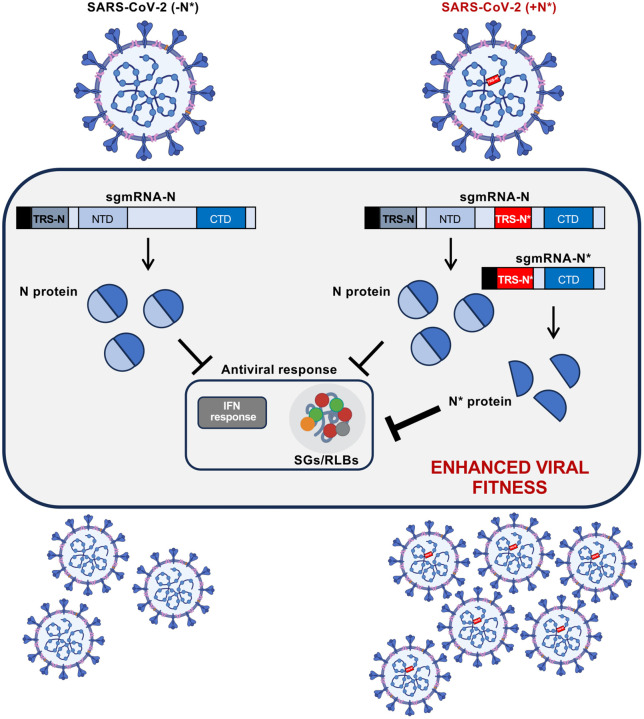
Enhanced fitness of SARS-CoV-2 by the expression of a truncated N protein that antagonizes antiviral responses. A triple mutation (GGG→AAC) internal to the N gene of SARS-CoV-2, which emerged with the ancestral B.1.1 lineage, created a novel transcription-regulating sequence (TRS-N*). TRS-N*, indicated as a red box on the viral genome, drives the production of a new subgenomic mRNA (sgmRNA-N*) encoding a truncated N protein (N*). The N* protein, referred to as “NiORF3” by Mears and colleagues and “N*M210” by Mulloy and colleagues, contains the C-terminal domain of N, which includes a dsRNA binding motif. N* interferes with host antiviral responses activated by viral dsRNA during infection, such as the production of interferon-β (IFNβ) following sensing by RIG-I [3] and the formation of ribonucleoprotein granules [4]. The thickness of the T-shaped lines is proportional to the inhibitory effect. Upregulation of N*, shown on the right part of the figure, increases antagonism of the antiviral response and enhances viral fitness, conferring a selective advantage. Mulloy and colleagues also reported the presence of N* in SARS-CoV-2 virions [4], supporting the idea that it may contribute, similarly to full-length N, to the packaging of the viral genomic RNA, as previously shown [10]. NTD, RNA sequence coding the N-terminal domain; CTD, RNA sequence coding the C-terminal domain. The TRS-N* is located in the Linker region between NTD and CTD. The black rectangle at the left end of the sgmRNAs represents the leader sequence common to all sgmRNAs. Partially created in BioRender. Sola, I. (2026) https://BioRender.com/zvqibul.

Mulloy and colleagues [4] provide more detailed insights into the mechanisms underlying the enhanced virus fitness conferred by the internal TRS-N*. The paper confirmed that the truncated N* protein, when overexpressed in cells, had a potent dsRNA binding activity and interfered with a variety of dsRNA-triggered cell antiviral responses [7,8]. They further showed in infected cells that N* expression broadly inhibited the formation of ribonucleoprotein granules, including stress granules (SGs) containing G3BP1, RNase-L dependent granules (RLB) [9] and P-bodies, via a dsRBM-dependent mechanism (Fig 1). Interestingly, the fitness advantage was lost in cells deficient in G3BP1/2 proteins. Because G3BPs are required for SG formation and are also part of RLBs, these results suggest that N* increased virus fitness, at least in part, by antagonizing SG or RLB formation [4].

While this novel effect was well documented in cell cultures, confirming granule inhibition in vivo in infected tissues remains an important next step. This discovery raises additional questions about the precise mechanisms underlying the antiviral activity of ribonucleoprotein (RNP) granules during SARS-CoV-2 infection. Addressing these questions is extremely challenging because RNP granules are complex and highly dynamic structures, with heterogeneous and poorly defined composition. This limited understanding significantly hampers the feasibility of functional studies that rely on knockout of specific factors required to suppress granule formation. Identifying RNAs included in RNP granules could reveal their potential effects on the translation regulation of viral and host RNAs.

Both papers [3,4] contribute to expanding our understanding of the complexity of SARS-CoV-2–host interactions. They highlight the biological impact of the emergence of a new sgmRNA encoding a truncated N protein, which can reshape the viral phenotype and influence virus adaptation. This supports the idea that mutations outside the spike protein may also contribute to virus evolutionary adaptation. Moreover, they shed light on the relevance of RNP granules in SARS-CoV-2 virus–host interactions. Further research is still needed to fully define granule composition, antiviral functions, and potential interactions among different RNP granules in infected cells, by combining current and emerging approaches in genetics, proteomics, transcriptomics, and imaging. This knowledge will be important for future efforts to identify new antiviral targets.

## Abbreviations

CS: core sequence
dsRBM: double-stranded RNA binding motif
IFN: interferon
N: nucleocapsid
RLB: RNase-L dependent granules
RNP: ribonucleoprotein
SGs: stress granules
sgmRNAs: subgenomic mRNAs
TRSs: transcription-regulating sequences
